# Predicting treatment response to systemic therapy in advanced gallbladder cancer using multiphase enhanced CT images

**DOI:** 10.1007/s00330-025-11645-7

**Published:** 2025-05-08

**Authors:** Ji Wu, Zhigang Zheng, Jian Li, Xiping Shen, Bo Huang

**Affiliations:** 1 Department of General Surgery, Suzhou Ninth Hospital Affiliated to Soochow University, Suzhou, China; 2 Department of Interventional Oncology, Renji Hospital, Shanghai Jiao Tong University School of Medicine, Shanghai, China; 3 Department of Radiology, The Affiliated Changshu Hospital of Nantong University, Suzhou, China; 4 Department of Hepatobiliary Surgery, Suzhou Municipal Hospital, The Affiliated Suzhou Hospital of Nanjing Medical University, Suzhou, China

**Keywords:** Gallbladder cancer, Treatment response, Combination therapy, Progress-free survival, Deep learning

## Abstract

**Background:**

Accurate estimation of treatment response can help clinicians identify patients who would potentially benefit from systemic therapy. This study aimed to develop and externally validate a model for predicting treatment response to systemic therapy in advanced gallbladder cancer (GBC).

**Methods:**

We recruited 399 eligible GBC patients across four institutions. Multivariable logistic regression analysis was performed to identify independent clinical factors related to therapeutic efficacy. This deep learning (DL) radiomics signature was developed for predicting treatment response using multiphase enhanced CT images. Then, the DL radiomic-clinical (DLRSC) model was built by combining the DL signature and significant clinical factors, and its predictive performance was evaluated using area under the curve (AUC). Gradient-weighted class activation mapping analysis was performed to help clinicians better understand the predictive results. Furthermore, patients were stratified into low- and high-score groups by the DLRSC model. The progression-free survival (PFS) and overall survival (OS) between the two different groups were compared.

**Results:**

Multivariable analysis revealed that tumor size was a significant predictor of efficacy. The DLRSC model showed great predictive performance, with AUCs of 0.86 (95% CI, 0.82–0.89) and 0.84 (95% CI, 0.80–0.87) in the internal and external test datasets, respectively. This model showed great discrimination, calibration, and clinical utility. Moreover, Kaplan–Meier survival analysis revealed that low-score group patients who were insensitive to systemic therapy predicted by the DLRSC model had worse PFS and OS.

**Conclusion:**

The DLRSC model allows for predicting treatment response in advanced GBC patients receiving systemic therapy. The survival benefit provided by the DLRSC model was also assessed.

**Key Points:**

***Question*** *No effective tools exist for identifying patients who would potentially benefit from systemic therapy in clinical practice.*

***Findings*** *Our combined model allows for predicting treatment response to systemic therapy in advanced gallbladder cancer.*

***Clinical relevance*** *With the help of this model, clinicians could inform patients of the risk of potential ineffective treatment. Such a strategy can reduce unnecessary adverse events and effectively help reallocate societal healthcare resources.*

**Graphical Abstract:**

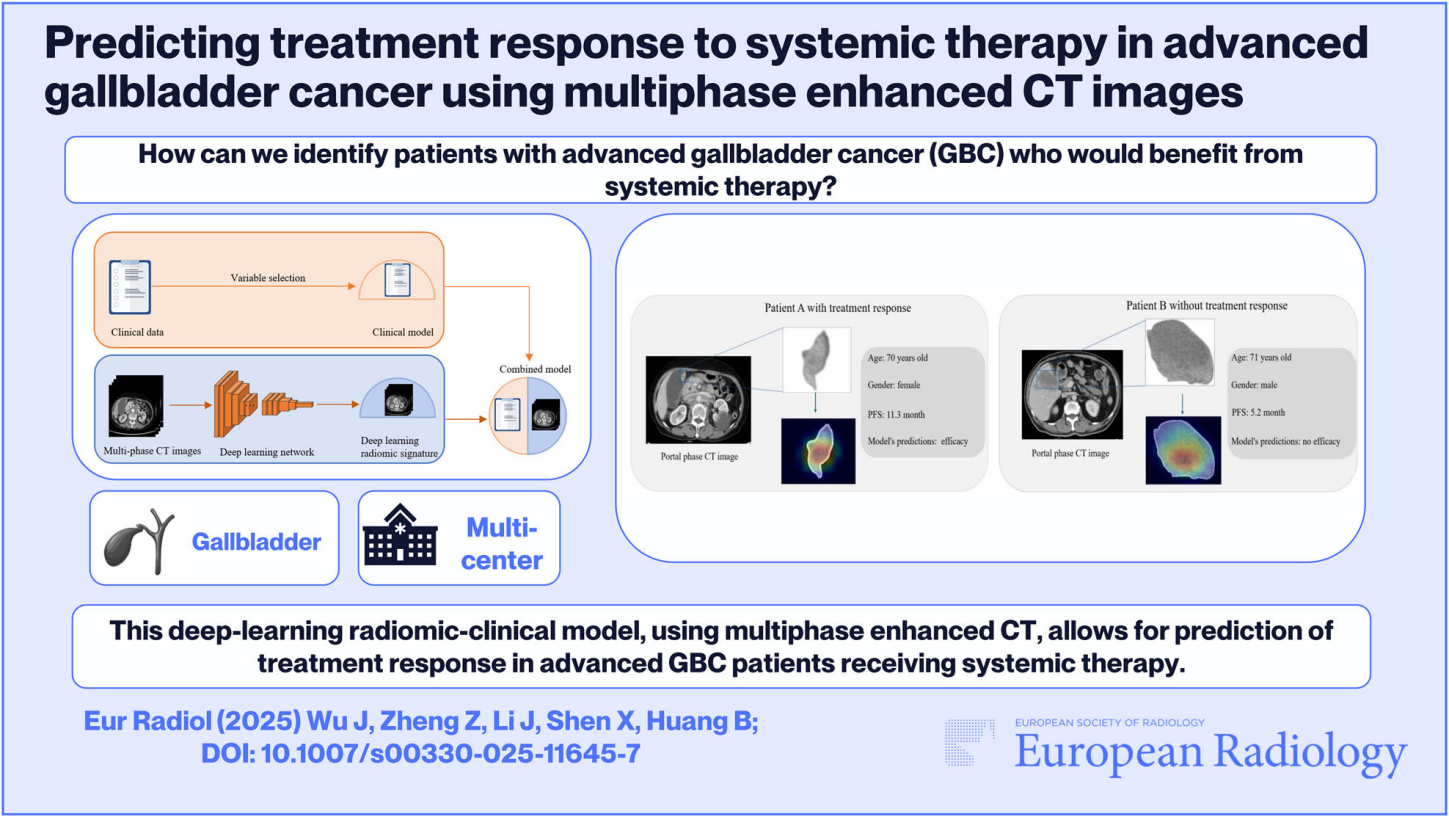

## Introduction

Gallbladder cancer (GBC) is a highly lethal hepatobiliary malignancy [1, 2]. The global incidence rate of GBC is on the rise. It is predominantly diagnosed at an advanced stage due to its atypical symptoms and aggressive tumor biology [3]. Surgical resection is not amenable for most GBC patients when diagnosed [4].

Systemic therapy has shown effectiveness in advanced GBC patients who are not surgical candidates [5]. Gemcitabine plus oxaliplatin (GEMOX) regimen has been the frequently preferred first-line standard of care. However, the efficacy of chemotherapy alone is still not satisfactory, and the mean overall survival (OS) is 8.3 months [6]. In recent years, immunotherapy using the anti-programmed cell death protein 1 (PD-1) antibody has shown promising results in advanced GBC [7, 8]. An improvement in OS was observed when anti-PD-1 antibody was added to the first-line chemotherapy regimen (gemcitabine plus platinum) [9]. However, different responses to combination therapy have been observed owing to intratumor heterogeneity. Patient selection based on reliable predictive biomarkers is crucial for maximizing efficacy and optimizing individual treatment strategies.

Nowadays, several tools have been proposed to predict treatment response [10–12]. However, most studies focused on clinicopathologic factors and predictive performances of their models were unsatisfactory. Additionally, it is often difficult to obtain adequate tumor tissue for molecular profiling, and it is an invasive procedure. Therefore, developing a noninvasive approach could be an intriguing topic for further investigation.

Radiomics, a well-validated method, provides new insights for non-invasive assessment of tumor heterogeneity and microenvironment by capturing subtle visual cues [13–15]. Furthermore, deep learning (DL) signature automatically derived by convolutional neural networks (CNN) have demonstrated remarkable capabilities in image discrimination [16, 17]. Despite this significant progress, few studies have yet reported successful prediction of treatment response to systemic therapy in advanced GBC patients using multiphase contrast-enhanced CT (CE-CT) images.

Hence, we aimed to develop and externally validate a deep learning radio-clinical signature (DLRSC) for noninvasively predicting the treatment response to systemic therapy by combining DL signature and clinical factors. In addition, the survival benefit provided by the final model was investigated.

## Materials and methods

### Study design

This retrospective multicenter cohort study has been reported in line with the STROCSS criteria [18]. We strictly followed the ethical guidelines of the 1975 Declaration of Helsinki. The Research Ethics Committee of institutions had approved it.

**Fig. 1:**
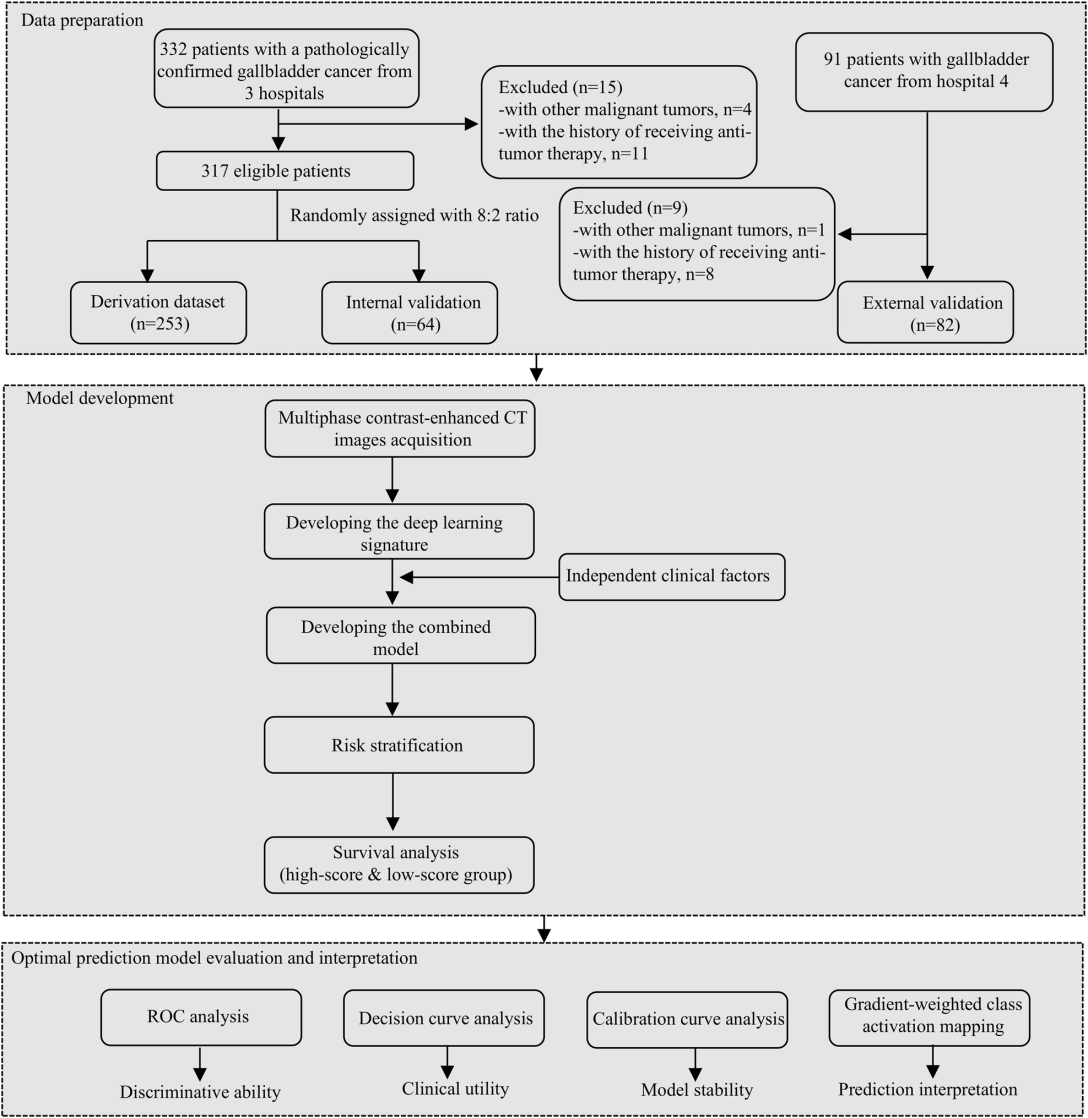
Flow diagram of study design. ROC, receiver operator characteristic curve

Advanced GBC patients from 4 hospitals between June 2017 and June 2023 were consecutively recruited for model development and evaluation. As shown in Fig. 1, 317 eligible patients from 3 hospitals were randomly divided into two groups (i.e., derivation dataset (*n* = 253) and internal test dataset (*n* = 64)) for model development and internal validation, with a ratio of 8:2 using a random number table. An independent external dataset that included data from 82 patients was used for external validation of the models. The inclusion criteria were (1) pathologically confirmed GBC before systemic treatment, (2) having at least one measurable lesion evaluated according to the Response Evaluation Criteria in Solid Tumors version 1.1 (RECIST v 1.1) and (3) complete clinical and CT image data. The patients with a history of receiving anti-tumor therapy or other malignancies simultaneously would be excluded.

### Clinical outcomes

Objective response rate (ORR) was defined for patients who achieved either complete response or partial response [19]. Tumor response to systemic therapy was evaluated with CT/MRI examination every 8–12 weeks according to RECIST version 1.1 [20]. The primary endpoint was the ORR of GBC to the anti-PD-1 antibodies plus GEMOX regimen. The additional endpoint of this study was progression-free survival (PFS), which was defined as the interval between the start of treatment and the date of progression of disease or last follow-up visit. OS was defined as the period between the initiation of treatment and death or the last contact.

### Selection of significant clinical predictors

Based on clinical knowledge and literature search, we collected potential clinical factors associated with therapeutic efficacy, including cancer antigen 19-9, carcinoembryonic antigen, alpha fetoprotein, and tumor size [21–23]. According to the 8th AJCC TNM staging system, cT (clinical tumor) stage, cN (clinical nodal) stage, and cM (clinical metastasis) stage were also recorded. Finally, we conducted multivariable logistic regression analysis to identify independent risk factors of poor therapeutic efficacy. In addition, a detailed description of tumor size measurement is available in Supplementary S1.

### Image acquisition

All individuals underwent baseline abdominal CE-CT examination before treatment initiation. Detailed scanning parameters are listed in Supplementary Table 1.

### Delineation of regions of interest

Tumor segmentation on three phase CE-CT images was performed using 3D slicer software (version 4.11) [24]. Two experienced radiologists who were unaware of clinical information manually labeled regions of interest (ROIs) using the “Level Tracing” function. The largest cross-sectional slice of lesion and its two nearest slices on the z-axis were delineated. The largest lesion was selected for downstream analysis if there were multiple tumors. Areas comprising air, large vessels and adjacent organs were excluded. The disputes between the two radiologists on the ROIs delineation were resolved after discussion. In our study, 50 patients’ images from the derivation dataset were selected at random for Dice Similarity Coefficient (DSC) calculation to evaluate the consistency of the manual segmentation. The average DSCs of the multiphase CT images were 0.91 ± 0.12 (arterial phase), 0.95 ± 0.03 (portal venous phase) and 0.92 ± 0.14 (delayed phase), respectively. All ROIs were reviewed by both radiologists.

### Data preprocessing

Peritumoral regions are the essential parts of the evaluation of tumor microenvironment. Tumor microenvironment showed close correlation with response to systemic therapy in patients with bile duct cancer [25, 26]. Therefore, for capturing quantitative information from the tumor microenvironment, tumor region masks were dilated with an expansion of 5 mm in all directions [27–29]. For the multiphase CT images, we adopted the generalized scale method to standardize the intensity of pixels [30]. Centers of the ROIs in each slice were selected for alignment. To ensure smooth convergence of model training, pixel values were normalized from [0, 255] to [0, 1] [31]. We reshaped images to a size of 224 × 224 pixels as the input data for the DL model.

Data augmentation was performed to improve the robustness of DL model before model training by applying a range of transformations (e.g., flipping and rotation) in the derivation dataset.

### Model development and evaluation

We introduced the pretrained Residual Network 18 (ResNet18) to develop the DL signature for predicting therapeutic efficacy [32, 33]. This transfer learning network is composed of four Blocks and each layer of convolution is followed by BatchNorm and ReLu activation functions. Sigmoid function was used as the final layer to produce the probabilistic predictions. We fine-tuned the DL signature on the derivation dataset using five-fold cross-validation. We employed early stopping to prevent overfitting. The parameter combinations were listed as follows: activation = ‘ReLu,’ optimizer = ‘Adam,’ classification function = ‘sigmoid,’ learning rate = ‘0.0002.’ Additionally, gradient-weighted class activation mapping (Grad-CAM) was performed to monitor the suspected tumor area detected by this network to make decisions regarding response or no-response. The DL signature was trained, tuned, and tested with Python software (TensorFlow library) (Intel Core i5-12600KF CPU, NVIDIA GeForce RTX 4060 GPU and RAM 32 GB). More information about model development is available in Supplementary S2.

We then built a combined model by incorporating selected clinical factors into DL signature using multivariable logistic regression algorithm in the derivation dataset. We conducted receiver operator characteristic curve (ROC) analysis to evaluate the predictive performance. Delong test was used to compare different ROC curves. The clinical utility and stability of models were assessed by decision curve (DCA) and calibration curve analysis, respectively.

For investigating the association of radiomic features specific to certain phases with treatment response, we respectively evaluated the predictive performance of arterial, portal venous and delayed phases CT images alone (Supplementary Fig. 1).

### Prognosis stratification

The model with the highest AUC value in the external test dataset was determined as the optimal model for further analysis. Based on the maximum value of Youden’s index, the whole dataset was divided into low and high-score groups. The PFS and OS for GBC patients were estimated using the Kaplan–Meier method. Survival time between two different scoring groups was compared using the log-rank test.

### Statistical analysis

We conducted data analysis with SPSS (v26.0), R (v4.0) and Python (v3.8.3) software. All significant tests were 2-sided and *p* < 0.05. Continuous data was described as the median ± interquartile range (IQR). The comparison of continuous variables was conducted by the Mann–Whitney U test or Student’s *t*-test. Categorical variables were compared by the Chi-square test. In our study, DCAs were assessed across a range of threshold probabilities from 10 to 40% [34, 35]. For instance, a threshold probability of 10.0% would indicate a willingness to perform 10 treatments to find one beneficiary. Within the evaluated range, the lower extreme of probabilities represented the greater willingness to receive combination therapy, whereas higher probabilities represented the less willingness for combination therapy despite a higher probability of effectiveness. Moreover, we estimated the sample size by using pmsampsize package of R software (R2cs = 0.28, parameters = 8), and then at least 246 cases were required for model development [36].

## Results

### Patient characteristics

**Fig. 2:**
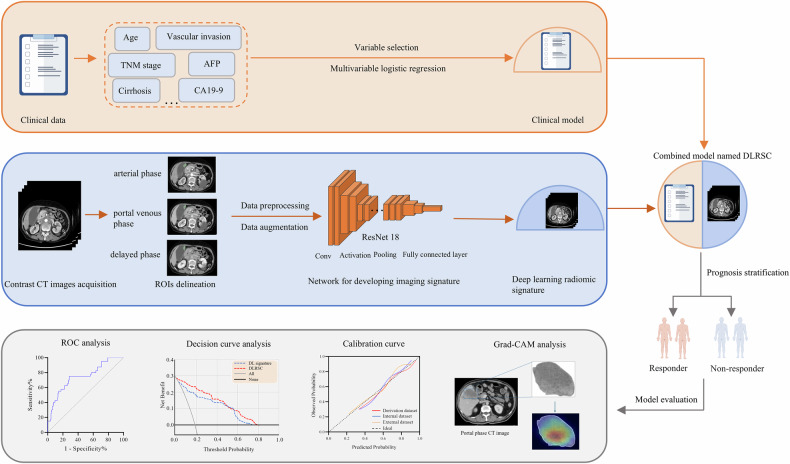
The workflow of model development. AFP, alpha-fetoprotein; CA19-9, carbohydrate antigen 19-9; ROI, region of interest; DLRSC, deep learning radiomic-clinical signature; ROC, receiver operator characteristic curve

The technological flowchart is presented in Fig. 2. In the present study, median PFS was 5.2 (IQR, 4.5) months for derivation dataset, 5.1 (IQR, 4.8) months for internal test dataset and 4.5 (IQR, 3.8) months for external test dataset, respectively (Table 1). Median OS was 10.1 (IQR, 8.3) months for derivation dataset, 9.5 (IQR, 10.7) months for internal test dataset and 10.9 (IQR, 10.1) months for external test dataset, respectively.

**Table 1:** Patient characteristics

Characteristics	Derivation dataset	Test datasets	
		Internal	External
No. patients	253	64	82
Age, year	64 (9)	62 (11)	65 (12)
Male gender	137 (54.2)	36 (56.3)	39 (46.4)
ACCI score	7 (5)	7 (6)	6 (5)
BMI, kg/m^2^	24.1 (4.5)	23.6 (4.1)	24.9 (5.2)
CA199, U/mL			
< 37	101 (39.9)	30 (46.8)	29 (35.4)
≥ 37	152 (60.1)	34 (53.2)	53 (64.6)
CEA, ng/mL			
< 100	198 (78.2)	50 (78.1)	69 (84.1)
≥ 100	55 (21.8)	12 (21.9)	13 (15.9)
AFP, ng/mL			
< 20	227 (89.7)	57 (89.1)	74 (90.2)
≥ 20	26 (10.3)	7 (10.9)	8 (9.8)
Tumor size, cm	5.2 (2.8)	5.3 (2.4)	4.6 (3.2)
cT stage			
T1-T3	144 (56.9)	35 (54.7)	45 (54.8)
T4	109 (43.1)	29 (45.3)	37 (45.2)
cN stage			
N0	61 (24.1)	19 (29.7)	24 (29.3)
N1-N2	192 (75.9)	45 (70.3)	58 (70.7)
cM stage			
M0	177 (70)	48 (75)	57 (69.5)
M1	76 (30)	22 (25)	25 (30.5)
Treatment response			
Partial response	63 (24.9)	14 (21.8)	22 (26.8)
Stable disease	55 (21.7)	16 (25)	24 (29.3)
Progression disease	135 (53.4)	34 (53.1)	36 (43.9)
Median PFS time, months	5.2 (4.5)	5.1 (4.8)	4.5 (3.8)
Median OS time, months	10.1 (8.3)	9.5 (10.7)	10.9 (10.1)

Quantitative values are median (IQR), and categorical variables are *n* (%)

*ACCI* age-adjusted Charlson Comorbidity Index, *BMI* body mass index, *CA199* carbohydrate antigen 199, *CEA* carcinoembryonic antigen, *AFP* alpha-fetoprotein, *PFS* progression-free survival, *OS* overall survival

### Assessment of the deep learning signature and DLRSC model

**Fig. 3:**
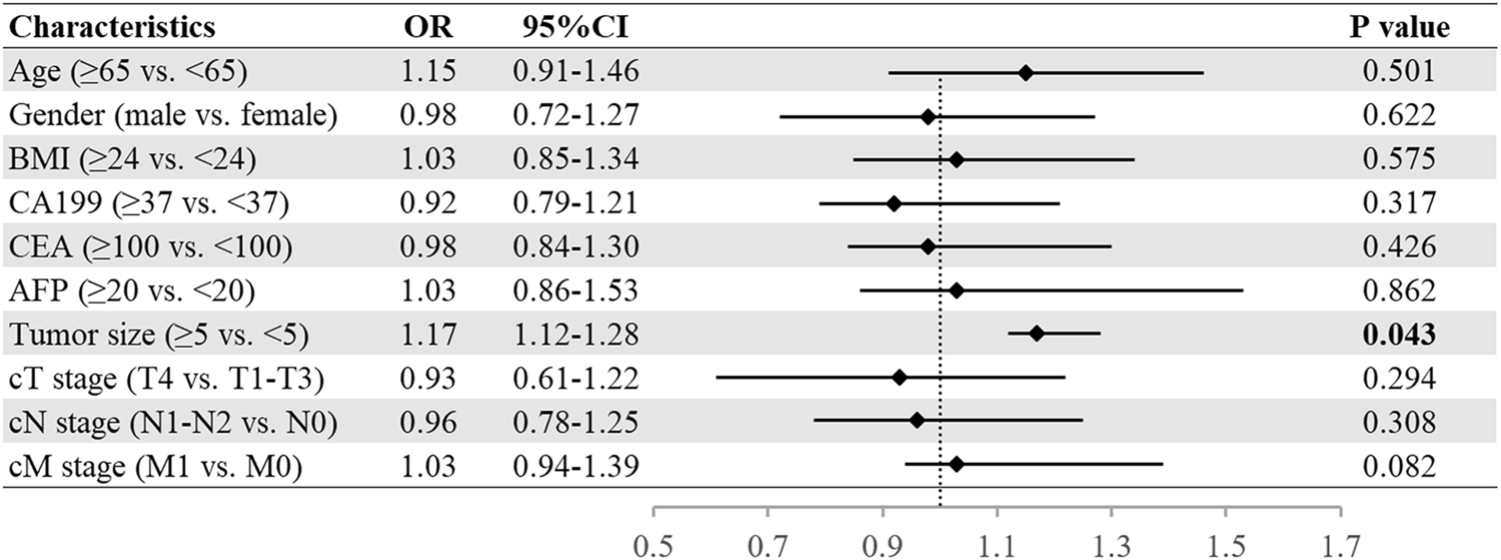
Forest plots for multivariable logistic regression analysis. They describe the association between each clinical factor and efficacy. The vertical line represents the value of no effect. Data are presented as the OR value with 95% CI. BMI, body mass index; CA19-9, carbohydrate antigen 19-9; CEA, carcinoembryonic antigen; AFP, alpha-fetoprotein; OR, odds ratio; CI, confidence interval

Multivariable logistic regression analysis results are shown in Fig. 3. Only tumor size (odds ratio (OR) = 1.17 (95% CI, 1.12–1.28), *p* = 0.043) was identified as an independent factor associated with therapeutic efficacy. Moreover, we reanalyzed the associations between clinical factors and efficacy in different patient subsets (Supplementary Fig. 3). The nature of the association between clinical factors and efficacy was consistent with our main findings.

**Fig. 4:**
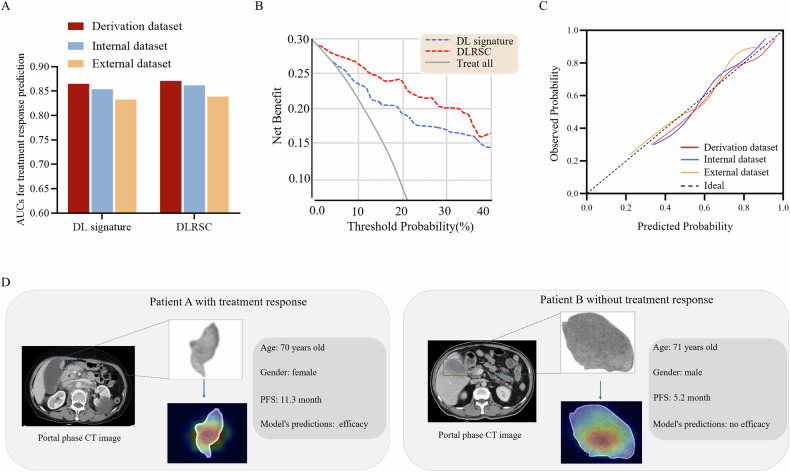
Model evaluation and interpretation. **A** AUCs for the DL signature and DLRSC model in the derivation and test datasets. **B** Decision-curve analysis illustrates the net benefit of the models for treatment selection. “Treat all” means “combination therapy for all.” Lower threshold probabilities for treatment selection indicate relatively greater priority to receive combination therapy for longer survival, whereas higher thresholds indicate relatively greater priority on avoiding combination therapy. For instance, a threshold probability of 10.0% indicates willingness to perform 10 treatments to find one beneficiary, whereas a threshold probability of 20.0% indicates willingness to perform 5 treatments to find one beneficiary. **C** Calibration plots of the DLRSC model in all datasets. **D** Representative examples of contrast-enhanced CT images (portal venous phase) and visualization of DLRSC model using Grad-CAM. AUC, area under the curve; DLRSC, deep learning radiomic-clinical signature; Grad-CAM, gradient-weighted class activation mapping

The predictive performance of the DL signature and DLRSC model are displayed in Fig. 4A. In the internal and external test datasets, the DL signature had AUCs of 0.85 (95% CI, 0.80–0.89) and 0.83 (95% CI, 0.80–0.86), respectively (Table 2). The DLRSC model yielded the best discriminative power, with AUCs of 0.86 (95% CI, 0.82–0.89) and 0.84 (95% CI, 0.80–0.87), respectively (Table 2). Delong test revealed that there was no significant difference between the AUCs of the DL signature and DLRSC model (*p* = 0.641). The DLRSC model with the highest AUC value in the external dataset was determined as the optimal model for downstream analysis. DCA plot revealed that the DLRSC model had a larger net benefit in the whole dataset (Fig. 4B). Calibration plots for the DLRSC model showed that this model was well calibrated in all datasets (Fig. 4C). A couple of confusion matrices were calculated (Supplementary Fig. 4).

**Table 2:** Predictive performance of the models for predicting the response of systemic therapy in advanced gallbladder cancer

Performance	Derivation dataset	Internal dataset	External dataset
DL signature			
AUC (95% CI)	0.87 (0.85, 0.90)	0.85 (0.80, 0.89)	0.83 (0.80, 0.86)
Sensitivity	0.826	0.809	0.778
Specificity	0.903	0.895	0.865
PPV	74.2	64.7	68
NPV	93.9	93.6	91.2
F1 score	78.2	73.3	72.3
Accuracy	0.885	0.859	0.841
DLRSC			
AUC (95% CI)	0.87 (0.84, 0.90)	0.86 (0.82, 0.89)	0.84 (0.80, 0.87)
Sensitivity	0.832	0.811	0.778
Specificity	0.910	0.902	0.883
PPV	75.7	70.5	70.8
NPV	94.5	95.7	91.4
F1 score	79.6	77.4	73.9
Accuracy	0.893	0.891	0.853

*DL* deep learning, *DLRSC* deep learning radiomic signature-clinical model, *AUC* area under of ROC curve, *CI* confidence interval

Moreover, activation maps were generated, and patient examples with different responses to systemic therapy for the actual use of the established DLRSC model are presented in Fig. 4D.

### Survival analysis

**Fig. 5:**
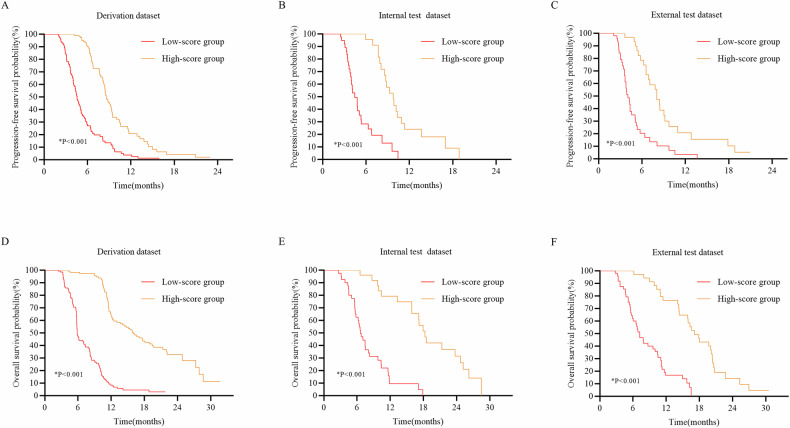
Kaplan–Meier survival curves for PFS (**A**–**C**) and OS (**D**–**F**) by the DLRSC model between high- (orange line) and low-score (red line) groups from the derivation and test datasets. Results revealed that low-score group patients had worse PFS and OS. PFS, progression-free survival; OS, overall survival; DLRSC, deep learning radiomic-clinical signature

Based on the best cutoff value, the threshold value of the DLRSC model was set as 0.536, and then the whole dataset was divided into low and high-score groups. Survival analysis was performed to evaluate the survival benefit provided by this model. Kaplan–Meier survival curves for PFS and OS are shown in Fig. 5A–F. There were significant differences in PFS and OS between two scoring groups stratified by the DLRSC model (log-rank *p* < 0.05). Patients in high-score group showed higher PFS and OS rates than those in low-score group for different subgroups (Supplementary Fig. 5).

## Discussion

The estimation of treatment responses to systemic therapy is crucial for making individualized therapies for advanced GBC patients. In this retrospective multicenter study, a novel combination model (DLRSC) for predicting treatment response was developed and externally validated using multiphase CE-CT images and clinical factors. The DLRSC model showed great predictive performance, with AUCs of 0.86 (95% CI, 0.82–0.89) and 0.84 (95% CI, 0.80–0.87) in the internal and external test datasets, respectively. This model showed great discrimination, calibration, and clinical utility. Grad-CAM analysis was performed to help clinicians better understand the predictive results. Furthermore, the survival benefit provided by the DLRSC model was also determined.

In previous studies, a few studies have investigated the predictors of response to systemic therapy in advanced GBC patients. These studies underwent a very limited external validation of its discriminatory ability and no evaluation of calibration. For example, Cheng et al retrospective analyzed 69 GBC patients and explored the predictors of immune therapy efficacy [11]. However, this single-center study was not validated using an independent external cohort.

In recent years, radiomics has made great achievements in tackling challenging clinical problems by capturing subtle visual cues and is increasingly gaining attention. It can extract plenty of quantitative features from radiological images [37]. What’s more, DL radiomics technique can automatically acquire feature representations from radiographic images on the basis of CNN, and it showed robust discrimination across large datasets [38]. To our knowledge, tumor heterogeneity and microenvironment showed close correlation with response to systemic therapy in patients with bile duct cancer [25, 26]. Meanwhile, tumor heterogeneity and microenvironment can be reflected on the radiological level [39–41]. Therefore, in the present study, we proposed an end-to-end DL signature derived from CNN and investigated the validity of the DL signature in predicting treatment responses and prognosis stratification. Analysis results revealed that the DL method may be sufficiently robust and generalizable for real-world applications. Furthermore, we found that portal venous phase CT images had better performance than arterial and delayed phase CT. It may be attributed to radiomic features related to tumor heterogeneity being more easily captured in the portal venous phase images. Notedly, portal venous phase CT images also exhibit significantly higher consistency of ROI segmentation compared to other phases. The better consistency of segmentations at portal venous phase seems to match well their informativeness. Even if the two factors may not be casually related, their synergistic effects in this phase may collectively contribute to the model’s great performance.

Multivariable logistic regression analysis revealed that only tumor size was identified as an independent factor associated with therapeutic efficacy. However, Delong test revealed that no significant differences between the AUCs of DL signature and DLRSC were found (*p* = 0.641). It suggested that tumor size may not significantly assist DL signature in efficacy prediction. It may be attributed to the fact that size measurement was based on imaging reports, and it has been captured by our model. We observed that the TNM staging showed no correlation with therapeutic efficacy in our study. It may be attributed to intratumor heterogeneity and tumor microenvironment. TNM staging system of gallbladder cancer was built based on tumor invasion, lymphatic metastasis status and distant metastasis. Not all tumors with the same TNM stage are equally responsive to combination therapy. Lesions with the same TNM stage may have significant biological differences, such as gene expression level and tumor-infiltrating lymphocytes, which have demonstrated a close association with efficacy [19, 42].

Furthermore, identifying patients who would benefit from subsequent combination therapy is essential for achieving favorable clinical outcomes. In our study, DCAs analysis revealed that the DLRSC model achieved a larger net benefit than DL signature. There was a positive impact on treatment decision-making when using the DLRSC model. At 10% probability threshold, the net benefit difference between the DL signature and DLRSC model was 0.022, indicating that 1/0.022 ≈ 46 treatments are needed for DLRSC model to find one additional beneficiary compared with the DL signature.

Our study has some limitations. First, although several methods have been employed to minimize the model overfitting, the risk of overfitting could not be fully eliminated owing to the relatively small sample size. This study should be validated in larger datasets from prospective studies. Second, manual delineation of ROI regions is influenced by subjective experience. An unsupervised segmentation method for tumor margins needs to be developed. In addition, DSC can reflect the spatial consistency of segmentation among different observers rather than the reproducibility of radiomics features. Although we minimized human variability through standardized segmentation protocols, potential noisy features may influence the model results. Third, a generalized scale method was first performed to balance images from different centers in the present study. However, inherent bias could be introduced due to the variability in image acquisition protocols across centers. Finally, although some confounding variables have been controlled for, other potential confounding variables, such as treatment variations, should be further evaluated in future studies.

## Conclusion

In conclusion, we developed and externally validated the DLRSC model for predicting treatment response to systemic therapy in advanced GBC patients by combining DL signature and clinical factors. This model provides valuable information for identifying potential patients who would benefit from combination therapy. In addition, the DLRSC model is effective in prognosis stratification in GBC patients. The generalizability of this model should be evaluated in future studies.

## Supplementary information

ELECTRONIC SUPPLEMENTARY MATERIAL

## Abbreviations

AUC: Area under the curve
CE-CT: Contrast-enhanced computed tomography
DCA: Decision curve analysis
DL: Deep learning
DLRSC: Deep learning radiomic-clinical signature
GBC: Gallbladder cancer
GEMOX: Gemcitabine plus oxaliplatin
Grad-CAM: Gradient-weighted class activation mapping
IQR: Interquartile range
OS: Overall survival
PD-1/L1: Programmed cell death protein 1/ligand 1
PFS: Progression-free survival
RECIST: Response Evaluation Criteria in Solid Tumors
ROIs: Regions of interest
