# Supplementary material for: Predicting treatment response to systemic therapy in advanced gallbladder cancer using multiphase enhanced CT images
Source: Eur Radiol. 2025 May 8;35(11):7410–20. doi: 10.1007/s00330-025-11645-7 (PMC12559086; doi:10.1007/s00330-025-11645-7)
Supplement: Supplementary file 1 — ELECTRONIC SUPPLEMENTARY MATERIAL [file 330_2025_11645_MOESM1_ESM.pdf]

Predicting treatment response to systemic therapy in advanced gallbladder cancer using multiphase enhanced CT images

ELECTRONIC SUPPLEMENTARY MATERIAL

Supplementary S1. Tumor size measurement

Radiologists independently measured the sizes of targeted lesions using the built-in ‘length’ tool of RadiAnt DICOM Viewer. The how-to manual of this software is available in an online website (https://www.radiantviewer.com/dicom-viewer-manual/index.html). Portal venous phase CT images were selected for tumor size measurement and the CT images were set to soft tissue window (W:350, L:40). The longest diameter of targeted lesion in the largest cross-sectional slice was recorded. The largest lesion was selected for evaluation if there were multiple tumors. The dispute would be settled after discussion. Tumor size was then included for univariate and multivariate logistic regression analysis as binary variables. We calculated the weighted kappa to explore the interobserver and intraobserver agreement when performing the size measurement of the lesions. Great agreement was observed in the present study (Supplementary Table 2).

Supplementary S2. Model development

In our study, ResNet18 was introduced to develop the DL radiomic signature. Squeeze-and-Excitation (SE) blocks have been empirically shown to improve network performance. We therefore added a channel-attention block before the first and after the last residual blocks. We described the architecture of SE-ResNet module in Supplementary Figure 2. To develop the DL signature, pre-processed multi-phase CT images were fed into the model as input. For our task, we first independently trained SE-ResNet18 model on the derivation dataset with treatment response as targeted labels. After training, SE-ResNet18 model can give a predicted probability of each category. A cut-off value determined using the Youden index was adopted to obtain the final predicted label. To obtain a relatively great model performance, we apply both a grid search and a random search strategy for selecting the optimal hyper-parameters (i.e., learning rate, optimizer). We then validate 30 different sets of hyper-parameter combinations. To determine the optimal parameter set, we conducted a total of 30 epochs to train the individual model corresponds to a specific hyperparameter set, and recorded the maximum prediction accuracy. The hyperparameter combination with the highest accuracy on the validation is chosen as the final parameter set for the model. A total of 100 epochs were conducted for this model and we chose the epoch with the maximum prediction accuracy on the validation as the final evaluated model. Finally, we conducted Grad-CAM analysis to monitor the suspected features of multiphase CT images detected by this network to make decisions regarding response or no-response.

Supplementary Table 1. CT protocols

Parameters	Hospital 1	Hospital 2	Hospital 3	Hospital 4
Vendors	SIEMENS Germany	SIEMENS Germany	GE Healthcare, USA	GE Healthcare, USA
Detector	64	128	64	64
Tube voltage (kVp)	120	120	120	120
Tube current t(mA)	260	250	280	250
Thickness (mm)	5	2	5	5
Contrast agent	Omnipaque (1.5 ml/kg)	Omnipaque (1.5 ml/kg)	Omnipaque (1.5 ml/kg)	Omnipaque (1.5 ml/kg)
Injection rate	3 ml/s	3 ml/s	2.5-3.5 ml/s	3 ml/s

Supplementary Table 2. Interobserver and intraobserver agreement κ Coefficients between two observers for tumor size measurement

Feature	Portal phase CT images	
	Interobserver agreement weighted κ (95% CI)	Intraobserver agreement weighted κ (95% CI)
Tumor size (≥5 vs.<5cm)	0.95 (0.92–0.98)	0. 98 (0.96–1.0)

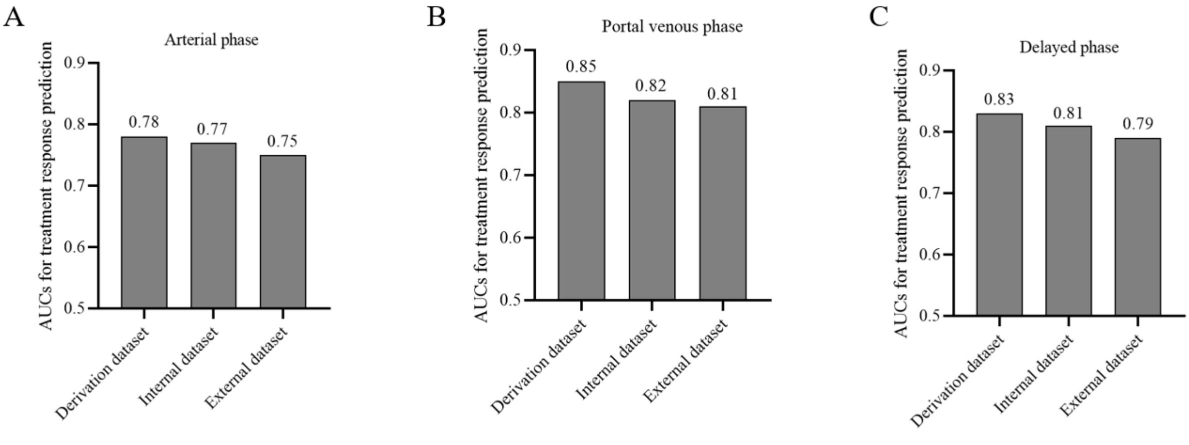

Supplementary Figure 1. Predictive performance of the arterial, portal venous and delayed phases CT images alone

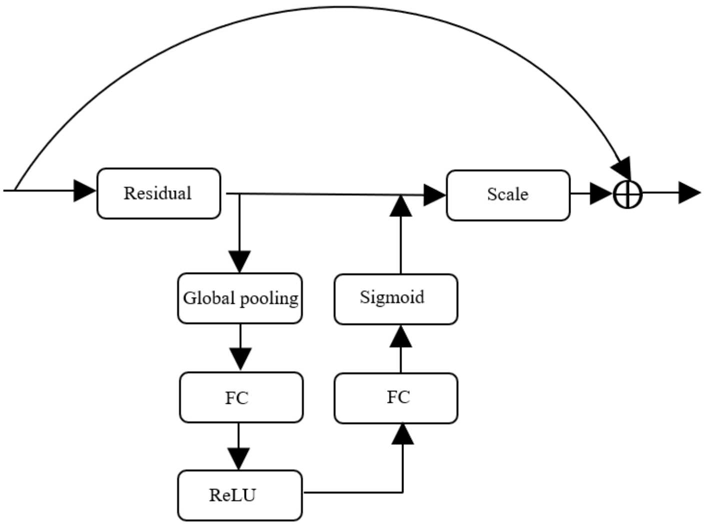

Supplementary Figure 2. The schema of the SE-ResNet module

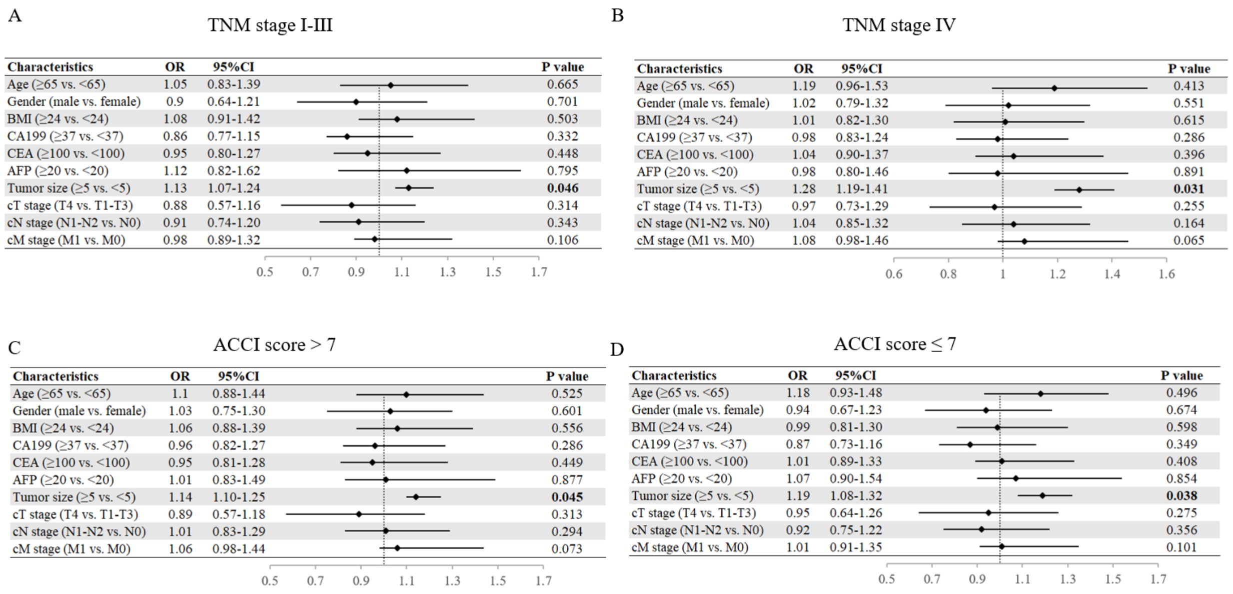

Supplementary Figure 3. Forest plots for multivariable logistic regression analysis in different patient subsets. Patients were divided into two groups based on the TNM stage or the median of ACCI score. They describe the association between each clinical factor and efficacy. The vertical line represents the value of no effect. Data are presented as the OR value with 95%CI. Abbreviation: ACCI, age-adjusted Charlson Comorbidity Index; BMI, body mass index; CA19-9, carbohydrate antigen 19-9; CEA, carcinoembryonic antigen; AFP, alpha-fetoprotein; OR, odds ratio; CI, confidence interval.

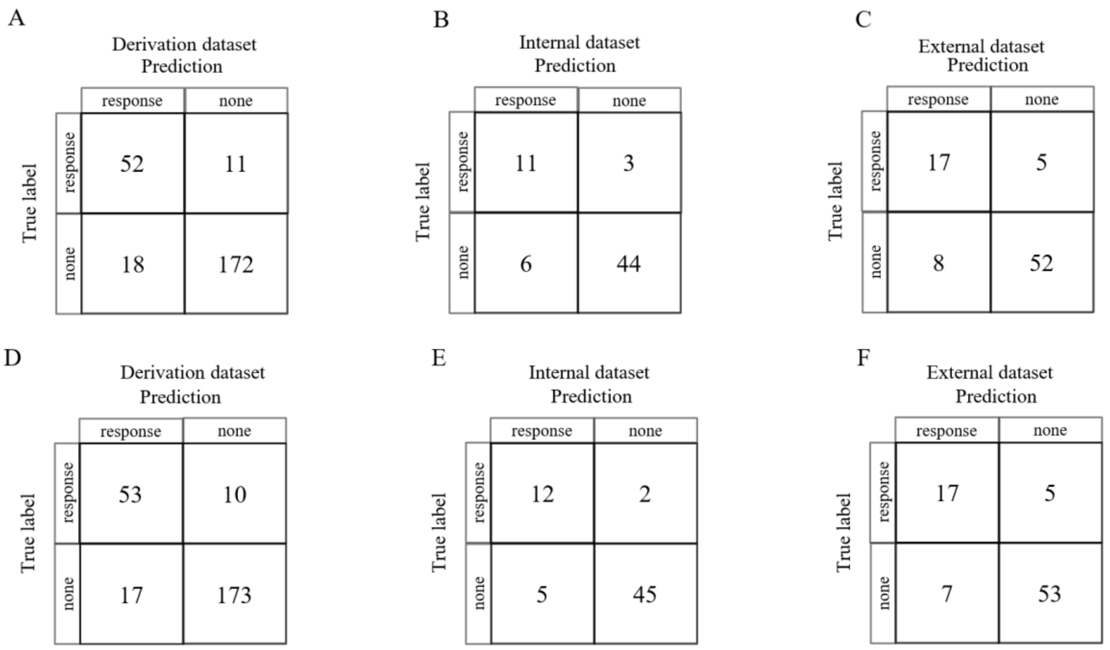

Supplementary Figure 4. Confusion matrixes of the DL signature(A-C) and DLRSC (D-F) models for predicting response of systemic therapy in advanced gallbladder cancer.

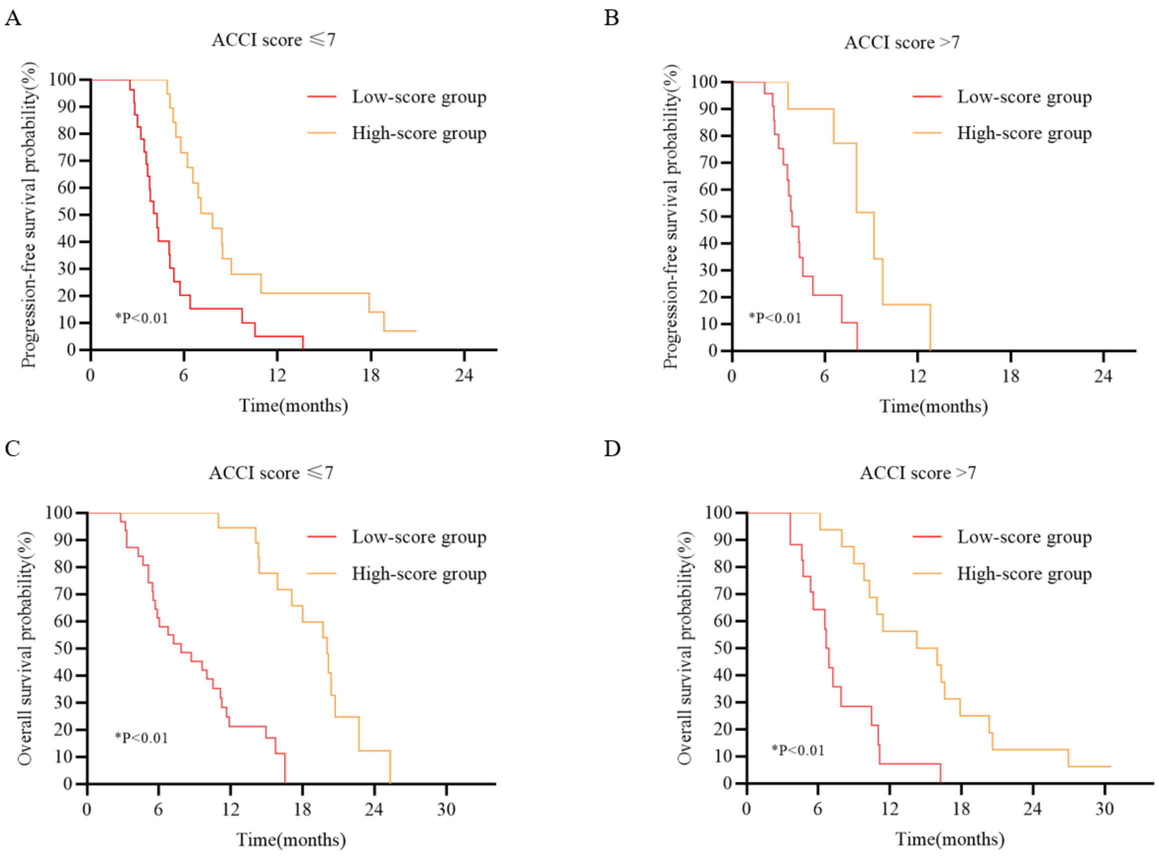

Supplementary Figure 5. Kaplan–Meier survival curves for PFS (A,B) and OS (C,D) by the DLRSC model between high- (orange line) and low-score (red line) groups from the external test dataset. Results revealed that low-score group patients also had worse PFS and OS. Abbreviations: PFS, progression-free survival; OS, overall survival; DLRSC, deep learning radiomic-clinical signature.
